# Enhancing early detection in youth psychiatry: neurobiological signatures of emotional- behavioral problems

**DOI:** 10.1192/j.eurpsy.2026.10331

**Published:** 2026-07-31

**Authors:** C. Colli

**Affiliations:** Fondazione IRCCS Ca’ Granda Ospedale Maggiore Policlinico, Milan, Italy

## Abstract

**Background:**

Clarifying the neurobiological basis of psychopathology requires characterizing the dynamic role of genetic and environmental influences and identifying biomarkers that can support neurobiologically informed models that goes beyond the strict categorical classification. In this context, we focus on the developmental period, a critical window for the emergence of mental health vulnerability, marked by heterogeneous and pleomorphic symptom expression.

**Methods and results:**

Different methodological approaches were applied to distinct subsamples of participants.

In a sample of 268 twins, classical univariate twin models were used to disentangle the contributions of genetic and environmental factors to schizotypy and hypomanic personality through the use of self report scales, that well characterizes subjective experiences. The roles of stressful life events and family relationship quality was also assesed.

Preliminary findings suggest a predominant role of environmental factors in modulating these vulnerability states, with a specific role of recent exposure to stressos and family climite in modulating presence of schizotypy.

In addition, a discordant-twin design—comparing pairs concordant or discordant for psychopathological conditions (13 twin pairs and 13 healthy controls)—was employed to evaluate task performance and cortical activation patterns associated with early psychotic susceptibility, particularly those related to cognitive–emotional interference.

Overall, our findings indicate that different types of emotional interference may reveal brain activity patterns that can serve as early markers of vulnerability or resilience to psychosis, primarily affecting basic facial perception rather than emotional appraisal mechanisms.

Moreover, we examined spontaneous narrative language in a transdiagnostic sample of 318 children and adolescents to identify dimensional linguistic profiles and explore their associations with internalizing and externalizing symptoms measured by the CBCL. Using k-means clustering, we identified three distinct linguistic subtypes. One subgroup, marked by pragmatic and fluency impairments, exhibited significantly higher internalizing and externalizing symptom scores, whereas a fluent and informative subgroup showed lower levels of psychopathology.

**Conclusions:**

Overall, these models were applied to both brain and clinical measures, underscoring the importance of adopting a neurodevelopmental approach that emphasizes early, non-specific manifestations alongside early alterations in subjective experience for improving the management of psychiatric disorders.

**Disclosure of Interest:**

None Declared

